# Metal Ion Effects on Aβ and Tau Aggregation

**DOI:** 10.3390/ijms19010128

**Published:** 2018-01-02

**Authors:** Anne Claire Kim, Sungsu Lim, Yun Kyung Kim

**Affiliations:** 1 Department of Neuroscience, Wellesley College, Wellesley, MA 02481, USA; akim22@wellesley.edu; 2 Brain Science Institute, Convergence Research Center for Diagnosis, Treatment and Care System of Dementia, Korea Institute of Science and Technology (KIST), Seoul 02792, Korea; sungsulim@kist.re.kr

**Keywords:** metal, tau, β-amyloid

## Abstract

Amyloid and tau aggregation are implicated in manifold neurodegenerative diseases and serve as two signature pathological hallmarks in Alzheimer’s disease (AD). Though aging is considered as a prominent risk factor for AD pathogenesis, substantial evidence suggests that an imbalance of essential biometal ions in the body and exposure to certain metal ions in the environment can potentially induce alterations to AD pathology. Despite their physiological importance in various intracellular processes, biometal ions, when present in excessive or deficient amounts, can serve as a mediating factor for neurotoxicity. Recent studies have also demonstrated the contribution of metal ions found in the environment on mediating AD pathogenesis. In this regard, the neuropathological features associated with biometal ion dyshomeostasis and environmental metal ion exposure have prompted widespread interest by multiple research groups. In this review, we discuss and elaborate on findings from previous studies detailing the possible role of both endogenous and exogenous metal ions specifically on amyloid and tau pathology in AD.

## 1. Introduction

Aided by the advances in medical technologies, the elderly population is increasing rapidly, and the world is now facing the ‘ageing era’, which comes with social issues like dementia. Alzheimer’s disease (AD) is the leading cause of dementia, affecting one-third of all people age 85 [1]. Ageing is recognized as the most important risk factor for AD, but a combination of genetic, environmental, and lifestyle factors also contribute to the onset of AD [2]. To reduce the risk of developing AD or help to treat it, substantial efforts have been made to identify the molecular causes of AD. Recently, accumulating evidence has suggested that metal dyshomeostasis in the brain is closely linked with age-related neurodegenerative disorders including AD.

**Figure 1:**
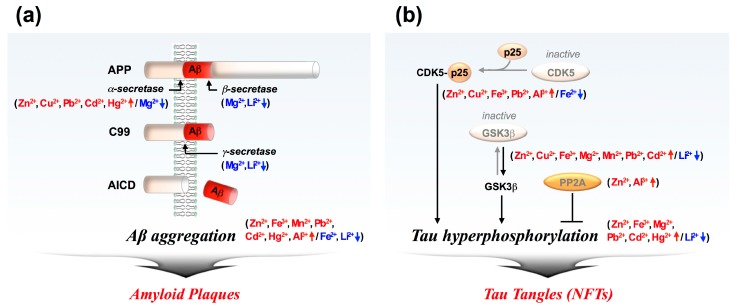
Metal ions effect on amyloid β (Aβ) and tau aggregation. (**a**) Amyloidogenesis [13]. Under normal conditions, Aβ is rarely produced in the brain, since amyloid precursor protein (APP) is cleaved by α- and γ-secretase. Under pathological conditions, APP is cleaved with β- and γ-secretase, generating neurotoxic Aβ peptide. Once the Aβ peptide is generated and secreted into the extracellular space, it spontaneously transforms into fibrils called amyloid plaques. Zn^2+^, Cu^2+^, Fe^3+^, Mn^2+^, Pb^2+^, Cd^2+^, Hg^2+^, and Al^3+^ induce amyloidogenic pathways and Aβ aggregation (red arrow). In contrast, Mg^2+^, Fe^2+^, and Li^2+^ reduce the formation of Aβ (blue arrow); (**b**) Tau pathology. In a nonpathological condition, tau is constantly phosphorylated and dephosphorylated for the maintenance of neuronal structure and function. Under pathological condition, tau is highly phosphorylated by diverse kinases such as cyclin-dependent kinase 5 (CDK5) [14] and glycogen synthase kinase-3beta (GSK-3β) [15]. Tau hyperphosphorylation could be maintained by a failure of activation of phosphatase like protein phosphatase 2A (PP2A) [16]. Hyper-phosphorylated tau aggregates into neurofibrillary tangles (NFTs). Zn^2+^, Cu^2+^, Fe^3+^, Mg^2+^, Mn^2+^, Pb^2+^, Cd^2+^, Hg^2+^, and Al^3+^ promote tau hyperphosphorylation and induce tau aggregation (red arrow). In contrast, Fe^2+^, and Li^2+^ reduce tau hyperphosphorylation (blue arrow). For further details and references on metal ions effect (see Table 1).

Metal ions are essential for life, playing important roles in the human body. In nature, nearly half of all proteins are metal-binding proteins called metalloproteins [3]. Protein-bound metal cations such as copper (Cu^2+^, Cu^+^), iron (Fe^3+^, Fe^2+^), magnesium (Mg^2+^), manganese (Mn^2+^), calcium (Ca^2+^), and zinc (Zn^2+^) are key elements for maintaining cell structure, regulating gene expression, mediating cell signaling as a second messenger, and catalyzing enzyme activities [4]. The brain, in particular, requires high levels of free metal ions in synaptic clefts as a modulator of synaptic transmission. Accordingly, metal dyshomeostasis directly causes neuronal dysfunction [5], leading to neuronal cell death [6]. Clinical studies have shown elevated levels of Cu, Fe, and Zn ions in post-mortem brain tissues of AD patients [7]. In addition to biometal dyshomeostsis, exposure to environmental heavy metals such as mercury (Hg^2+^), cadmium (Cd^2+^), lead (Pb^2+^), aluminum (Al^2+^), and lithium (Li^+^) is neuro-toxic [8], leading to the activation of AD pathology (Figure 1).

AD pathology involves a wide variety of neurotoxic pathways such as abnormal protein aggregation, mitochondria dysfunction, reduced synthesis of neurotransmitters, inflammation, and oxidative stress in the brain. Among them, accumulation of amyloid β (Aβ) and tau aggregates is considered the most dominant etiologic paradigm of Alzheimer’s pathology [9]. Aβ is a 39 to 43 amino acid long peptide generated through abnormal proteolysis of the amyloid precursor protein (APP) by β- and γ-secretases. Once the Aβ peptide is secreted, it spontaneously transforms into neurotoxic oligomers and fibrils, which damage neuronal cells [10] (Figure 1a). In contrast, tau is an abundant protein in the brain, supporting neuronal structures and functions [11]. Under pathological conditions, tau is abnormally hyper-phosphorylated, and the hyper-phosphorylated tau aggregates into fibrils called neurofibrillary tangles (NFTs) (Figure 1b). Abnormal tau tangles are accumulated in neurons, causing neuronal toxicity and neurodegeneration [12]. Metal dyshomeostasis has been suggested as a strong neurotoxic candidate that induces changes in Aβ and tau aggregation. Here, we will review the pathological roles of metal ions in the alteration of amyloidogenesis and tau pathology.

## 2. Essential Biometal Ions

### 2.1. Zinc

Zinc is an important metal ion in the body that regulates synaptic transmission, brain development, and immune function [17,18,19]. Physiological concentrations of zinc in the human brain range from 56.7 to 75.9 μg/g [20]. Clinical studies report an increase of brain levels of zinc at a significantly higher range of 62.0 to 89.9 μg/g in AD patients compared to age-matched control subjects. Zinc dyshomeostasis in the brain is linked with multiple neurotoxic implications as it can potentially trigger neuronal injury and severely worsen the pathogenesis of neurodegenerative diseases. In addition to studies detailing zinc-induced neuronal injury and cell death, researchers have also been looking at how an imbalance of brain zinc levels can serve as a contributing factor to Aβ and tau pathology in AD. The primary mechanism of zinc on Aβ pathology remains elusive and controversial [21]. One possible hypothesis states that micromolar zinc binding to a high affinity site on the Aβ peptide induces a conformational change of the partially folded monomeric form of the Aβ protein to a more unfolded amyloidogenic conformation [22,23,24]. Related to binding affinity of Zn to Aβ peptide, the *K_d_* values of Zn to Aβ 40 are 20 ± 14 and 7 ± 3 μM at high and low concentrations of Zn, respectively [25]. As a result, the hydrophobic surfaces of the Aβ protein are more exposed, leading to enhanced hydrophobic interactions and Aβ protein aggregation [26]. The overall final product of the metal-induced conformational change of the Aβ peptide is demonstrated to be a toxic non-fibrillar oligomeric form different from the conformation of fibrils free of zinc [27]. However, in the case where the millisecond kinetics of fibril growth is fast, zinc has been shown to slow additional fibril formation by interfering with the compatibility between the co-existing zinc-bound fibrils and the zinc-free fibrils [28,29]. Furthermore, zinc has been demonstrated to induce neuronal cell death and toxicity directly involved in AD by mediating the blockage of Aβ ion channels at the surface of the cell [25,30,31,32]. Moreover, abnormally high concentrations of zinc increase the resistance of Aβ peptides to α-secretase cleavage and, therefore, promote an increase in Aβ content marked by further oxidative damage and inflammatory responses [33]. Moreover, it has been found that zinc can inhibit the α-secretase cleavage activity of APP, resulting in elevated β- and γ-secretase processing of APP and a further increased generation of extracellular Aβ plaques. Previous studies have further demonstrated that synaptic signaling pulses of zinc at micromolar concentrations both promote aggregation of the Aβ40 peptide and stabilize the generated toxic oligomers in vitro and in vivo [34]. Specifically, it has been found that zinc can inhibit the α-secretase cleavage activity of APP, resulting in elevated β- and γ-secretase processing of APP and a further increased generation of extracellular Aβ plaques [35]. Another study has agreeably demonstrated zinc-induced aggregation of Aβ peptides in vitro [36]. Recently, zinc has also been demonstrated to induce tau hyperphosphorylation by activating the glycogen synthase kinase-3beta (GSK-3β) and inactivating phosphatase like protein phosphatase 2A (PP2A) [18,35,37]. However, whether zinc-mediated tau hyperphosphorylation involves the GSK-3β kinase remains controversial as some studies show that the GSK-3β kinase is not activated and only PP2A is inactivated under zinc-induced tau hyperphosphorylation conditions [38]. Additionally, an in vitro study has found that PP2A is not involved in zinc-promoted tau hyper-phosphorylation [39]. Taken together, these findings suggest that zinc is involved in inducing both Aβ and tau aggregation. Future studies can more closely examine the specific pathways involved in zinc-mediated tau hyperphosphorylation as well as consider the involvement of environmental zinc in Aβ and tau pathology in AD.

### 2.2. Copper

Copper is an essential element in the body that serves as a cofactor for various intracellular enzymes and proteins, with a physical concentration range of 3.1 to 5.1 μg/g in the brain [40,41]. When metal ions are in dyshomeostasis in the brain, they undergo a shift from being tightly bound to proteins to adopting a loosely bound, chelatable form [42]. The transformation that metal ions undergo when they are at imbalanced concentrations in the brain is consistent with the decreased levels of copper bound to proteins in the soluble region of the AD cerebral cortex and the increased content of chelatable forms of copper in the same AD cerebral cortex brain region [43]. Moreover, the loosely bound, free flowing copper in the cytoplasm is demonstrated to be the leading factor behind the increased copper content in the blood serum-plasma of AD patients [44]. The binding constant of copper to APP is as low as 0.013 ± 0.005 μM [45]. The intimate link between copper dyshomeostasis and AD established through several meta-analyses studies has, in recent years, influenced researchers to investigate how copper can potentially hold adverse effects on both Aβ and tau aggregation in AD. The perturbation of copper ion status from being tightly bound to proteins to being loosely free in the cytoplasm holds significant biochemical consequences. Specifically, copper has been shown to aggregate Aβ peptides in AD by specifically increasing APP cleavage activity [46]. Interestingly, however, a recent study has shown that copper can reduce the accumulation of Aβ plaques by increasing the APP cleavage activity of the α-secretase enzyme [47]. In addition to its role in Aβ aggregation, copper is also reported to be involved in tau aggregation. Currently, copper is known to induce tau hyper-phosphorylation by activating the cyclin-dependent kinase (CDK)5/p25 complex and GSK-3β kinase [46,48,49]. However, an in vivo study involving oral copper administration to AD transgenic mice has contrastingly revealed a marked increase in CDK5/p25 complex activity but no significant activation of the GSK-3β kinase, implying that the GSK-3β kinase may not necessarily be involved in tau hyperphosphorylation [46]. Moreover, with a *K_d_* value of 0.5 μM, treatment of copper to a tau R3 peptide induces conformational changes in which the peptide adopts a monomeric α-helical structure and β-sheet structure [50]. The formation of these two distinct structures facilitates the self-aggregation and assembly of the tau protein into paired helical filaments in vitro [51]. Although copper has consistently been shown to induce tau aggregation, the specific pathways through which it hyperphosphorylates tau remains unknown. In addition, the effects of copper on Aβ aggregation remain unclear. Despite considerable progress made in characterizing the effects of copper on Aβ and tau pathology in AD, the specific pathways through which copper mediates tau hyperphosphorylation remain complex and require further comprehensive understanding. In addition, forms of copper are also present in the environment and reports have highlighted its toxic physiological effects. Therefore, future studies could also be directed at investigating the role of environmental copper on Aβ and tau pathology.

### 2.3. Iron

As a vital metal ion in the body, iron serves to regulate neurotransmitter synthesis, mitochondrial function, and myelin development [52,53,54]. Physiological concentrations of iron in the brain range from 216 to 272 μg/g and are reportedly higher at a range of 288 to 322 μg/g in the brains of AD patients [55,56]. When present in excess, iron might induce detrimental pathological disruptions or drive cellular death. Iron’s toxic characteristics along with its ability to mediate the formation of radicals make it a major facilitator for neurodegenerative diseases. Although the etiology of iron in neurodegeneration remains unclear, investigators have sought to understand the potential effects of iron on both Aβ and tau aggregation in AD. With respect to Aβ pathology, iron has been shown to induce Aβ aggregation in vitro [57]. In opposition, however, an in vivo study has demonstrated that Aβ aggregation was reduced following iron treatment [58]. Furthermore, manifold evidence implicates iron’s role in the hyperphosphorylation of the tau protein both in vitro and in vivo by activating the CDK5/p25 complex and GSK-3β kinase [59,60]. As iron is present throughout the brain in various redox states, whether ferrous iron (Fe^2+^) or ferric iron (Fe^3+^) induces or reverses tau aggregation requires more substantial evidence [59,61,62]. With reference to the considerable number of studies reviewed, it is evident that iron modulates both Aβ and tau aggregation. Iron, however, is also found in the environment in the form of dietary intake. Therefore, examining the effects of excess environmental iron consumption on Aβ and tau aggregation can serve centrally to the overall understanding of iron-mediated Aβ and tau pathology in AD.

### 2.4. Magnesium

Magnesium is an important biometal ion that regulates synaptic plasticity, muscle function, protein synthesis, and ribosome structure stability [63,64]. Within the brain, magnesium concentrations range from 620 to 680 μg/g and in AD patients the range is significantly lower at levels of 540 to 625 μg/g [65]. Clinical studies have associated the reported magnesium deficiency with severe pathology and neurobehavioral perturbations. Given the deleterious implications of magnesium deficiency on neuronal health, new areas of investigation have been looking at how magnesium dysmetabolism plays a role in Aβ and tau pathology in AD. The effects of magnesium on Aβ aggregation remain disputable as some studies show that magnesium reduces Aβ plaques while others report an elevation of Aβ plaques following magnesium treatment. Specifically, magnesium has been shown to lower the expression of the β-secretase enzyme that cleaves APP, reducing the generation of Aβ plaques [66]. The biometal ion has also been demonstrated to enhance the APP cleavage activity of the α-secretase enzyme, reducing the formation of Aβ plaques [67]. Interestingly, magnesium has also been found to stabilize the γ-secretase enzyme and elevate its APP cleavage activity [68]. Though the various effects of magnesium on Aβ aggregation have been established and widely investigated, the role magnesium plays in tau aggregation remains to be further examined. Recent studies, have demonstrated that magnesium induces the aggregation of tau paired helical filaments (PHF_tau_) in vitro [69]. In contrast, administration of magnesium to an AD transgenic mouse model has been shown to increase the phosphorylation of the GSK-3β kinase at the Serine 9 phosphorylation site which reduces the hyperphosphorylation activity of the tau protein [70]. Studies to date have evidently demonstrated magnesium’s influence on amyloid and tau pathology in AD, both arguably increasing but also elucidating the aggregation of amyloid and tau proteins. As magnesium is also found in the form of dietary intake in the environment, future studies could examine the therapeutic potential magnesium supplementation embodies in alleviating AD pathogenesis.

### 2.5. Manganese

As an important biometal ion in the body, manganese serves as a cofactor for intracellular enzymes, regulates brain development, and controls metabolic and immune functioning [71]. Manganese concentrations in postmortem tissues range from 2.0 to 2.5 μg/g and are reported to be elevated in the brains of AD patients [72]. Upon reports that highlight the risk of excess manganese for neurotoxicity and disruptions to cellular processes, investigators have been critically examining the implications of manganese dyshomeostasis on Aβ and tau pathology observed in AD. The elevation of manganese levels has been shown to accelerate the aggregation of Aβ proteins by down-regulating enzymes that regulate the degradation of extracellular Aβ deposits [73]. A down-regulation of the enzymes can further lead to an increased accumulation of Aβ plaques. In addition, manganese has also been shown to induce tau hyperphosphorylation in vitro by first activating the GSK-3β kinase which further hyperphosphorylates the tau protein [74]. Although the aforementioned findings point to manganese’s effects on Aβ and tau aggregation, there are very few limited studies supporting this conclusion. Therefore, further extensive research is needed to fully confirm manganese’s role in Aβ and tau pathology in AD. Although manganese is an essential metal ion in the body, epidemiological studies have shown that manganese, when present at high concentrations in drinking water, carries neurotoxic effects with the capability of perturbing neurodevelopment, damaging DNA, as well as mediating neurotoxicity. Examining how fetal exposure to environmental forms of manganese can potentially influence the development and progression of Aβ and tau pathology serves as a therapeutic potential in the field of AD research.

## 3. Environmental Metal Ions

### 3.1. Lead

Lead is a naturally occurring toxic heavy metal ion found all throughout the environment in the form of contaminated air, soil, and water [75]. The correlation between lead exposure and cognitive decline in humans revealed through several longitudinal and cross-sectional studies point to the importance of understanding how exposure to lead can potentially be implicated in Aβ and tau aggregation in AD. Early lead exposure is demonstrated to be involved in the aggregation of the Aβ protein. Neonatal exposure to lead has been shown to result in an increased expression of the *APP* gene in vivo that persisted into adulthood and was followed by an elevation in both APP cleavage activity and the generation of extracellular Aβ deposits [76]. Similarly, another study has shown that early lead exposure can induce significant alterations to the brain that are maintained until old age where eventually increased Aβ protein expression becomes prominent [75]. Recently, lead has also been shown to aggregate the tau protein. Exposure of wild-type mice to lead-contaminated drinking water at birth resulted in increased levels of hyperphosphorylated tau in vivo when the mice were old [77]. Lead-mediated tau hyperphosphorylation is driven by the increased activity of the CDK5/p25 complex as well as the GSK-3β kinase [77,78,79,80]. Altogether, these findings highlight that lead not only induces Aβ aggregation by increasing APP cleavage activity but also induces tau aggregation via the CDK5/p25 complex and GSK-3β kinase.

### 3.2. Cadmium

Cadmium is a poisonous heavy metal ion found in contaminated forms within the environment [81]. With recent epidemiological studies reporting cadmium’s severe neurotoxic properties, many researchers have focused on understanding how cadmium may affect the aggregation of the Aβ and tau proteins in AD. Previous findings indicate that cadmium is involved in the aggregation of the Aβ protein. By inhibiting the activity of the α-secretase enzyme, cadmium increases APP cleavage activity which results in increased deposition of extracellular Aβ plaques [82]. Cadmium has also been demonstrated to reduce the expression of an Aβ degrading enzyme, resulting in an increased accumulation of Aβ plaques [82]. In addition, cadmium treatment to in cell models has resulted in a direct dose-dependent increase in Aβ plaque levels [83]. Furthermore, in cell models and in vivo studies show that cadmium indirectly induces tau hyperphosphorylation by activating the GSK-3β kinase, resulting in increased hyperphosphorylated tau [83,84]. Increased aggregation of tau fragments exposed to cadmium has also been demonstrated in a previous in vitro study [81]. Taken together, these findings illuminate cadmium’s role as an environmental pollutant, indirectly mediating both Aβ and tau aggregation.

### 3.3. Mercury

Mercury is an environmental heavy metal ion that is commonly ingested in the forms of contaminated air, food, and water [85,86]. Diversified studies from the past have continually highlighted the debilitating consequences of mercury including motor and cognitive disturbance as well as the neurotoxic effects. In recent years, investigators have been examining the effects of mercury contamination on neurodegenerative diseases, more specifically Aβ and tau aggregation in AD. Mercury exposure has been shown to promote the increased accumulation of extracellular Aβ deposits in an in cell model [87]. A recent study, however, showed that treatment of an in cell model with mercury led to decreased levels of APP, possibly resulting in reduced accumulation of Aβ deposits [88]. In vitro studies have also demonstrated that mercury is involved in promoting the aggregation of the tau protein [87,89]. Although substantial findings emphasize mercury’s role in mediating tau aggregation, further clarification on whether mercury promotes or reduces the aggregation of the Aβ protein is essential.

### 3.4. Aluminum

Aluminum is the most widely distributed metal ion in the environment and is commonly incorporated in everyday life. Nonetheless, mounting evidence has highlighted the debilitating neurotoxic consequences of chronic aluminum exposure on the central nervous system. In addition, recent studies have suggested that aluminum exposure might also be associated with neurobehavioral changes. As aluminum has been detected in both the Aβ plaques and neurofibrillary tangles, researchers have also centered their focus on examining the effects of aluminum on both AB and tau pathology in AD. Currently, it is known that aluminum treatment to neuronal cultures has resulted in a marked accumulation of Aβ aggregates in vitro [90]. An additional study has reported a similar pattern of an increase in Aβ aggregation following aluminum exposure [91]. In addition to its involvement in Aβ pathology, aluminum is also reported to strongly promote tau aggregation by driving the reduction of PP2A activity along with increasing CDK5 kinase levels and activating the GSK3-β kinase [92,93]. Therefore, as evidenced in the previous studies aforementioned, aluminum is shown to be involved in inducing both the aggregation of Aβ and tau proteins.

### 3.5. Lithium

Lithium is a pharmaceutical metal ion regarded as a primary form of treatment for mood disorders. As lithium has also been shown to have neuroprotective effects for various neurodegenerative diseases, previous studies have looked at how lithium can potentially elucidate Aβ and tau pathology in AD [94,95,96]. Studies have demonstrated that lithium reduces the formation of extracellular Aβ deposits in the brain in vivo by lowering the activity of β- and γ-secretase cleavage of APP [97]. Another study has similarly found that treatment of lithium lowers the accumulation of extracellular Aβ deposits in an in cell model [98]. The reduction of tau hyperphosphorylation following lithium administration has been reported as well. Lithium can suppress the levels of phosphorylated tau by directly inhibiting the activity of the GSK-3β kinase enzyme both in vivo and in cell based models [99,100]. Though substantial evidence points to lithium’s role in inhibiting tau hyperphosphorylation, whether lithium reduces or does not alter Aβ aggregation deserves to be further investigated.

## 4. Discussion

Alzheimer’s disease (AD) is a devastating neurodegenerative disease characterized by a severe decline in cognitive function and neuropathological alterations, and its etiology has been associated with various biological and environmental risk factors, including aging. Increasing evidence suggests that AD pathogenesis, specifically the aggregation of amyloid and tau, is also influenced by metal ions. In this respect, our review has looked at previous studies examining the effects of two groups of metal ions (i) essential biometal ions in the body and (ii) metal ions found in the environment. Biometal ions, though physiologically important, when imbalanced, can act as harmful cofactors in the pathogenesis of AD. In addition, the ubiquity of environmental toxic metal ions and their easy ability to spread make them a global health threat. Moreover, certain pharmacological environmental metal ions, including lithium, can hold great potential in therapeutic approaches to mitigating AD pathogenesis. Despite the considerable progress made in demonstrating the role of metal ions on amyloid and tau aggregation, the specific pathways and mechanisms remain much more complex than reviewed here. Subsequently, the metal ion concentrations incorporated in the discussed studies may not fully reflect both the concentrations of biometal ions in the human brain and metal ions in the environment sufficient to exert effects on amyloidogenesis and tau pathology in AD. Therefore, we believe future studies can look to closely examine the specific neurochemical processes that precede the activation of the signaling kinases central in this review.

## Figures and Tables

**Table 1:** Metal ion effects on amyloid beta and tau aggregation.

Metal Ion	Metal Conc.	Aβ agg.	Study Type	Model	Mechanism	Metal Conc.	Tau agg.	Study Type	Model	Mechanism
Zn	100–250 μM	+	In cell	Human tau (1N4R) transfected 1C9 clonal CHO cell [35]	Inhibits α-secretase enzyme activity	100–250 μM	+	In cell	Human tau (1N4R) transfected 1C9 clonal CHO cell [35]	Inactivates PP2A/Activates GSK-3β kinase
	25–50 μM	+	In vitro	Synthesized human Aβ42 peptide [22], Aβ40 peptide [23,24]	Induces Aβ conformational change and aggregation	10–300 μM	+	In cell	N2a cell [38]	Inactivates PP2A
						100–400 μM	+	In cell	SH-SY5Y cell [39]	Induces tau hyperphosphorylation
	20 nM/10 μM	−	In vitro	Synthesized human Aβ42 peptide [28,29]	Inhibits Aβ fibrillization	50–500 μM	+	In vitro	Rat cortical neurons [18]	Activates GSK-3β kinase
	25 μM	+	In vitro	Radiolabeled/unlabeled human Aβ_40_ peptide [36]	Induces Aβ aggregation	10–100 μM	+	In vitro	Rat hippocampal slices [37]	Inactivates PP2A
Cu	5–200 μM	+	In cell	CHO cell [47]	Increases α-secretase enzyme activity	400 μM	+	In cell	SH-SY5Y cell [49]	Activates GSK-3β kinase
	250 ppm	+	In vivo	3xTg-AD mouse [46]	Increases APP cleavage activity	25 μM	+	In vivo	APP/PS1 mouse [48]	Activates GSK-3β kinase
						250 ppm	+	In vivo	3xTg-AD mouse [46]	Activates CDK5/p25
Fe	1 mM	+	In vitro	Radiolabeled/unlabeled human Aβ_40_ peptide [57]	Induces Aβ aggregation	50 μM	+	In cell	SH-SY5Y cell [59]	Activates CDK5/p25 & GSK-3β kinase
						20 μM	−	In cell	Rat hippocampal neurons [62]	Disrupts CDK5/p25
						1 mmol/L	+	In vitro	PHF_tau_ fractions [61]	Induces tau aggregation
	1 mM	−	In vivo	*D. melanogaster* Aβ system [58]	Impedes Aβ aggregation	10 mg/mL	+	In vivo	APP/PS1 tg mouse [60]	Activates CDK5/p25 & GSK-3β kinase
Mg	0–0.4 mM/1.2–4.0 mM	−	In cell	N2a cell [67]	Increases α-secretase enzyme activity	5 mM	+	In vitro	Sarkosyl-insoluble fractions of PHF_tau_ prepared from post-mortem AD brain [69]	Induces PHF_tau_ aggregation
	5 mM	−	In cell, In vivo	SH-SY5Y cell [68]	Stabilizes γ-secretase enzyme activity	50–200 mg/kg	+	In vivo	Streptozotocin-induced sporadic AD rat [70]	Increases GSK-3β phosphorylation at Ser9
	~910 mg/kg	−	In vivo	APPswe/PSEN1dE9 tg mouse [66]	Reduces β-secretase enzyme activity					
Mn	0–400 μM/60 mg/kg	+	In cell, In vivo	N2a cell, APPswe/PSEN1dE9 tg mouse [73]	Decreases Aβ degradation enzyme	100–500 μM	+	In cell	PC12 cell [74]	Activates GSK-3β kinase
Pb	0.2%	+	In vivo	C57BL/6 mouse [75]	Increases Aβ protein expression	0.2%	+	In vivo	C57BL/6 mouse [77]	Increases tau hyperphosphorylation
						1.5 mg/kg	+	In vivo	*M. fascicularis* primate [78]	Increases CDK5 levels
	200 ppm	+	In vivo	Long-Evans rat [76]	Increases *APP* gene expression	0.2%	+	In vivo	hTau tg mouse [79]	Increases GSK-3β & CDK5 kinase activity
						0.1%	+	In vivo	Wistar rat [80]	Increases GSK-3β & CDK5 kinase activity
Cd	1–100 μM	+	In cell	SN56 cell [83]	Increases Aβ deposits	1–100 μM	+	In cell	SN56 cell [83]	Activates GSK-3β kinase
	2.5 mg/kg	+	In vivo	APP/PS1 mouse [82]	Inhibits α-secretase enzyme activity, Decreases of Aβ degradation enzyme	3.8 μM	+	In vitro	Tau fragment R3 (third repeat of microtubule -binding domain) [81]	Accelerates tau aggregation
						3.75–6 mg/kg	+	In vivo	ICR mouse [84]	Activates GSK-3β kinase
Hg	36 nM–18 μM	+	In cell	SH-SY5Y cell [87]	Increased accumulation of Aβ plaques	36 nM–18 μM	+	In cell	SH-SY5Y cell [87]	Induces tau hyper-phosphorylation
	5–20 μM	+	In cell	SH-SY5Y cell	Decreases APP levels and reduces Aβ aggregation [88]	2.25–15 μM	+	In vitro	Tau fragment R2 (second repeat of microtubule -binding domain) [89]	Induces tau aggregation
Al	1 mM	+	In vitro	Rat cortical neurons [90]	Induces Aβ aggregation	0.4–1.6 mg/kg	+	In vivo	Wistar rat [92]	Inhibits PP2A activity and accelerates tau aggregation
	1.88 × 10^5^ mol/L	−	In vitro	Human Aβ_40_ peptide [91]	Induces Aβ aggregation	100 mg/kg	+	In vivo	Wistar rat [93]	Increases CDK5 levels and induces tau aggregation
Li	-	−	In cell	HEK293 cell [96]	Reduces Aβ aggregation	300–600 mg/kg	−	In cell, In vivo	HEK293 swAPP_751_, PDAPP mouse [98]	Reduces tau hyperphosphorylation
	0.18 mmol	−	In vivo	APP/PS1 mouse [97]	Decreases β-/γ-secretase enzyme activity	100 mg/mL	−	In vivo	APP/PS1 mouse [99]	Phosphorylates GSK-3β kinase

Metal ion induces (+) or reduces (−) Aβ and tau aggregation.
